# Positive secular trend in excess body weight in adolescents: A comparative study of 2007 and 2017/2018 data

**DOI:** 10.1371/journal.pone.0310452

**Published:** 2024-12-23

**Authors:** Fernanda Ulsula de Souza, Clair Costa Miranda, Mateus Augusto Bim, Luiz Rodrigo Augustemak de Lima, Isadora Gonzaga, Gaia Salvador Claumann, Thais Silva Beltrame, André de Araújo Pinto, Andreia Pelegrini

**Affiliations:** 1 Center for Health and Sports Sciences, Santa Catarina State University, Florianópolis, Santa Catarina, Brazil; 2 Institute of Physical Education and Sports, Federal University of Alagoas, Maceió, Alagoas, Brazil; 3 Department of Physical Education, Roraima State University, Boa Vista, Roraima, Brazil; Amazonas State University, BRAZIL

## Abstract

**Background:**

This study aimed to compare the prevalence of excess weight in adolescents living in Florianópolis, Santa Catarina, Brazil, from 2007 to 2017/2018 and assess associations with physical activity level, screen time, muscle strength, and cardiorespiratory fitness.

**Methods:**

Two cross-sectional surveys were conducted with adolescents (14 to 19 years old). The body mass index was calculated from measurements of body weight (kg) and height (m^2^). Information on sociodemographic variables (sex, age, and economic level), screen time, and physical activity was collected using questionnaires. Motor tests were used to measure cardiorespiratory fitness and muscle strength.

**Results:**

A positive secular trend in excess weight was observed in adolescents between 2017/2018 and 2007. Boys who spent more than 4h a day on screen-based activities and those with inadequate cardiorespiratory fitness were 1.96 and 3.03 times more likely, respectively, to have excess weight in 2017/2018 than in 2007. Boys (OR = 1.77) and girls (OR = 1.74) with inadequate muscle strength were more likely to have excess weight in 2017/2018 than their peers in 2007. Furthermore, boys and girls with adequate cardiorespiratory fitness had 64% and 90% lower chances, respectively, of having excess weight in 2017/2018 than in 2007.

**Conclusions:**

A decade of observation revealed a positive secular trend in excess weight in adolescents, with significant associations with muscle strength, cardiorespiratory fitness, and screen time.

## Introduction

Obesity is considered one of the leading public health problems worldwide [1]. This chronic condition is characterized by accumulation of body fat at excessively high levels that can be detrimental to health [2], contributing to the emergence of cardiovascular and metabolic diseases and impairing quality of life and mental health [3]. Furthermore, obesity is associated with lower levels of physical fitness, affecting both cardiorespiratory and muscular capabilities [4].

Numerous studies point to an increase in the prevalence of excess weight (overweight and obesity) in children and adolescents [5, 6]. A combined analysis of different studies conducted around the world between 1975 and 2016, encompassing about 31.5 million children and adolescents, showed that the prevalence of obesity increased in both sexes, escalating from 0.7% to 5.6% in girls and from 0.9% to 7.8% in boys [7]. In Brazil, an increase in overweight and obesity was observed in different regions of the country [5, 8] and found to be linked to genetic, environmental, and behavioral factors [3, 6].

The increasing prevalence of excess weight can be attributed to lifestyle, a multifaceted concept encompassing behavioral, environmental, and psychosocial factors, as well as the food environment and environmentally driven reductions in physical activity [9]. Low levels of physical activity [10], excessive screen time [6], increased consumption of ultra-processed and high-calorie foods [11, 12], sleep deprivation, and exposure to endocrine-disruption chemicals can contribute to weight gain, influenced by genetic susceptibility, age, and sex [9]. Consequently, guidelines advocate for children and adolescents to engage in at least 60 minutes of moderate to vigorous physical activity daily [13], and to limit screen time [13]. Various cutoff points are recommended, such as 2–3 hours per day [14] and 4 hour per day [15], reflecting concerns over the health risks associated with sedentary behavior and screen time.

Furthermore, studies indicate that physical activity levels have decreased among adolescents in various countries [16], coinciding with an increase in screen time [17–19]. Specifically, within the context of adolescents in Florianópolis, where this study was conducted, previous research has shown a 28.1% reduction in physical activity levels [20]. Moreover, 52.2% of adolescents were insufficiently active, and 50.1% reported more than 4 hours of daily screen time [21]. These findings are concerning, given that physical activity and screen time are related to muscular fitness and cardiorespiratory fitness [13]. Besides, 12.5% of adolescents from Florianópolis were overweight, and 3.2% were obese [21].

Temporal analyses of excess weight in adolescent populations are crucial for designing and implementing actions in different segments of society, particularly for the development of public policies and interventions aimed at encouraging behavioral changes and mitigating this health problem over the years [22]. In view of the foregoing, this study aimed to examine the temporal trend in excess weight in adolescents living in Florianópolis, Santa Catarina, Brazil, over a decade (2007 to 2017/2018) and verify for possible associations with the level of physical activity, screen time, muscle strength, and cardiorespiratory fitness.

## Materials and methods

### Study design

The study consisted of two cross-sectional surveys conducted over a 10-year period. The data for the first study were collected between July 1st and December 20th, 2007, by researchers from the Federal University of Santa Catarina (UFSC). The data for the second study were collected between August 1st, 2017, and May 30th, 2018, by researchers from the State University of Santa Catarina (UDESC). One of the researchers responsible for the data collection in 2007 also coordinated the second survey (2017/18). Research procedures were approved by the Human Research Ethics Committee at the UFSC in 2007 (protocol No. 372/2006) and at the UDESC in 2017/2018 (protocol No. 2.172.699). Participating schools, parents/guardians, and the adolescents themselves provided written consent for their participation in the study.

### Setting

The study was conducted in the urban area of the city of Florianópolis, a capital city located in the center-east of Santa Catarina State, Brazil. Florianópolis has a territorial area of 674,844 km^2^ and an estimated population of 537,213 people in 2022 [23]. In 2010, there were 32,573 individuals aged from 15 to 19 years [23]. The GDP per capita was R$ 41,885.53 in 2020, and the average monthly salary of formal workers was 4.5 minimum wages in 2021 [23]. The percentage of the population with a monthly per capita income of less than half the minimum wage was 24.6% in 2010 [23].

### Participants

The sample comprised adolescents of both sexes, aged 14 to 19 years, attending state public schools in Florianópolis. The inclusion criteria for both surveys were being aged 14 to 19 years, being regularly enrolled in high school at one of the selected state education schools for the study, and being present in the classroom at the time of data collection. The exclusion criteria were being outside the specified age range and having a physical or clinical condition that prevented the completion of physical tests or the questionnaire. The sample was calculated based on the school census of the respective years. In the year 2007, the number of adolescents attending state high schools in the municipality of Florianópolis was 12,741, and in 2017/18, it was 10,192. From a finite population, with a confidence level of 1.96, a tolerable error of four percentage points, and an estimated prevalence of 50% (unknown outcome), 10% of the number of students in the sample was added to the result to mitigate potential losses due to incorrect or incomplete questionnaire responses or dropout during data collection [24], calculations resulted in a minimum sample of 631 adolescents in 2007 and 624 adolescents in 2017, both composed of different adolescents. The total sample was distributed proportionally among the five regions defined by the Municipal Health Secretariat (Center, Mainland, East, North, and South). Only the largest school in terms of number of students of each municipal region participated in the study.

For participant selection in both surveys, classes were randomly selected by sortition in each school until the number of students needed within each region was reached. Data collection took place on school days during class hours, with authorization from the classroom teacher. All students in the selected classes were invited to participate (cluster sampling).

### Variables

The body mass index (BMI) (dependent variable) was calculated by dividing body weight (kg), measured to the nearest 100 g by using a Plenna^®^ digital scale, by the square of height (m), measured to the nearest 0.1 cm by using a Sanny^®^ portable stadiometer [25]. BMI was categorized into normal weight (underweight + normal weight) and excess weight (overweight + obesity) according to sex-and age-specific cutoffs for BMI values [26, 27]. These cutoff points were established based on samples from various countries, including Brazilian children and adolescents. Furthermore, these cutoff points are widely used in studies involving this population [6].

The independent variables were physical activity, sedentary behavior, and physical fitness. The daily level of physical activity was measured using the International Physical Activity Questionnaire (IPAQ)–Short Form, which has been validated for Brazilian adolescents [28]. Adolescents who met the recommendations for moderate-to-vigorous intensity physical activity were classified as active, whereas those who did not meet the recommendations were classified as insufficiently active [29].

Screen time was self-reported through a questionnaire applied in classroom, where the adolescent recorded the time (minutes per day) spent on screen-based activities, such as watching television, playing video games, and using the computer. Screen time was calculated individually for each electronic device. The total screen time was calculated for weekdays (Monday to Friday) and weekend days (Saturday and Sunday). The daily average screen time was calculated by adding the total screen time of weekdays and the total screen time of weekend days and dividing by seven [30]. The cut-off point for excessive screen time was 4 h a day [15].

Muscle strength was evaluated using the handgrip test [25]. Handgrip strength was measured using a Jamar^®^ dynamometer (2 kg resolution). Both hands were evaluated alternately, with two attempts per hand. Each maximum muscle contraction lasted 5 seconds, with an interval of one minute between each measurement [31]. The best score (kg) of each hand was added to obtain the overall score (Right-hand score + Left-hand score = Overall score). Muscle strength was classified as adequate (normal + high) and inadequate (low) according to sex- and age-specific cut-offs [31].

Cardiorespiratory fitness (CRF) was assessed using the modified Canadian Aerobic Fitness Test (mCAFT) [25]. The mCAFT consists of 8 stages where participants step up and down a two-step bench for 3 minutes, with a 20-second break between each stage. Before starting the test, the participant´s age is recorded to calculate their 85% effort heart rate using the equation 220 minus age. As the test progresses through each stage, the stepping cadence increases. The test concluded when the participant can no longer maintain the correct cadence, experiences pain or discomfort preventing further participation, or reaches their 85% effort heart rate threshold. CRF was obtained by the equation 17.2 + (1.29 × VO_2_ in the last completed step)– 0.09 × Body weight in kg) − (0.18 × Age in years) (24). CRF values are typically classified as excellent, very good, good, fair, and poor, but we chose to group individuals with good, very good, and excellent cardiorespiratory fitness into adequate cardiorespiratory fitness and those with fair and poor cardiorespiratory fitness needs into inadequate cardiorespiratory fitness.

Sex (male and female) and age (complete years) were self-reported through a questionnaire applied in class. Economic level was estimated according to the Brazilian Criteria of Economic Classification [32, 33]. Total scores were transformed into a tercile distribution. For evaluation of sexual maturity, data were collected in the 2007 survey using the criteria proposed by Tanner [34] and in the 2017/2018 survey using an instrument developed by Adami and Vasconcelos [35], which is based on stages of pubic hair growth. Stage 1 represents the infantile phase (prepubertal); stages 2, 3, and 4 represent the maturational process (pubertal); and stages 5 and 6 indicate the mature adult stage (post-pubertal). Both scales were applied by undergraduate students in Physical Education and Physical Education professionals pursuing a Master’s or Doctorate degree.

### Statistical analysis

Descriptive statistics (mean, standard deviation, and frequency distribution) and inferential statistics were used. Analyses were stratified by sex and survey year, and differences in proportions were compared between groups. Logistic regression analyses were conducted considering normal weight as reference category, excess weight as output variable, and screen time (<4 h a day vs. ≥4 h a day), physical activity (active vs. insufficiently active), muscle strength (adequate vs. inadequate), and cardiorespiratory fitness (adequate vs. inadequate) as exposure variables. These analyses were adjusted for economic level and sexual maturity. The percentage change (delta) in the outcome variable between the two surveys was calculated as the difference between the prevalence of outcome in 2017/2018 and 2007, divided by the 2007 prevalence, and multiplied by 100. The significance level for all analyses was set at 5%. Analyses were performed using IBM SPSS Statistics 20.0 and BioEstat 5.3. Dataset is available on the Figshare repository (dx.doi.org/10.6084/m9.figshare.24881565).

## Results

The sample comprised 2172 adolescents (2007, *n* = 1146; 2017/2018, *n* = 1026). After exclusion of individuals who did not provide answers to all study variables, the total number of participants (2007 + 2017/2018) was 1533 adolescents. The 2007 survey included 563 adolescents, 344 girls (61.1%) and 219 boys. The 2017/2018 survey included 970 adolescents, 497 boys (51.2%) and 473 girls. The descriptive data, including age, body mass, height, body mass index (BMI), economic level, handgrip strength, cardiorespiratory fitness (CRF), physical activity (min/day), and screen time, are presented in Table 1 (male) and Table 2 (female). These tables also provide comparative values between 2007 and 2017/18. In 2007, the majority of boys were active (56.6%), whereas girls were insufficiently active (54.4%). Most spent more than 4 hours per day on screen-based activities (89.5% of boys and 93.0% of girls), had adequate handgrip strength (95% of boys and 96.2% of girls), and exhibited adequate cardiorespiratory fitness (86.3% of boys and 87.8% of girls).

**Table 1:** Descriptive analysis of male adolescents and comparison of values between surveys (2007-2017/18) in Florianópolis, Santa Catarina, Brazil.

Variable	Male		p-value
	2007 (n = 219)	2017/18 (n = 497)	
	$\bar{x}$ (dp)	$\bar{x}$(dp)	
Age (years) ‡	16.10 (1.16)	16.57 (1.06)	< 0.001
Body mass (kg) †	63.05 (10.46)	67.01 (13.14)	< 0.001
Height (cm) †	172.59 (7.21)	177.12 (7.06)	0.553
BMI (kg/m²) ‡	21.16 (3.28)	21.31 (3.74)	0.992
Economic level (score) ‡	20.05 (3.88)	31.29 (6.55)	< 0.001
HGS total (kg) †	85.24 (17.86)	80.16 (17.06)	0.790
CRF (mL/kg.min) †	52.16 (4.14)	49.77 (4.72)	0.020
Physical activity (min/day) ‡	90.02 (112.75)	68.18 (78.77)	< 0.001
Video gaming (min/day) ‡	221.43 (200.77)	112.79 (160.78)	< 0.001
Computer use (min/day) ‡	80.80 (94.83)	125.98 (176.62)	0.791
TV watching (min/day) ‡	196.42 (154.42)	118.63 (136.21)	< 0.001
Total screen time (min/day) †	498.65 (292.59)	357.41 (275.90)	0.768
	**n (%)**	**n (%)**	**p-valor**
**Physical activity level** ^#^			0.007
Insufficiently active	95(43.4%)	270 (54.3%)	
Active	124 (56.6%)	227 (45.7%)	
**Screen time** ^#^			< 0.001
<4 h	23 (10.5%)	197 (39.6%)	
>4 h	196 (89.5%)	300 (60.4%)	
**Muscle strength** ^#^			0.001
Adequate	208 (95.0%)	431 (86.7%)	
Inadequate	11 (5.0%)	66 (13.3%)	
**Cardiorespiratory fitnes**s^#^			< 0.001
Adequate	189 (86.3%)	330 (66.4%)	
Inadequate	30 (13.7%)	167 (33.6%)	

$\bar{x}$: mean; (dp): standard deviation; BMI: body mass index; HGS: handgrip strength; CRF: cardiorespiratory fitness; TV: television.

^†^ Independent t-test;

^‡^ Mann-Whitney U test;

^#^ Chi-Square Test

**Table 2:** Descriptive analysis of female adolescents and comparison of values between surveys (2007-2017/18) in Florianópolis, Santa Catarina, Brazil.

Variable	Female		p-value
	2007 (n = 344)	2017/18 (n = 473)	
	$\bar{x}$(dp)	$\bar{x}$(dp)	
Age (years) ‡	16.05 (1.10)	16.40 (1.04)	< 0.001
Body mass (kg) †	53.77 (8.45)	57.78 (11.33)	< 0.001
Height (cm) †	161.12 (6.00)	165.57 (6.21)	0.442
BMI (kg/m²) ‡	20.67 (2.92)	21.03 (3.69)	0.002
Economic level (score) ‡	19.15 (4.00)	30.28 (6.16)	< 0.001
HGS (kg) †	56.61 (10.74)	51.59 (11.09)	< 0.001
CRF (mL/kg.min) †	41.98 (3.09)	39.78 (3.36)	< 0.001
Physical activity (min/day) ‡	79.87 (94.91)	41.73 (70.71)	< 0.001
Video gaming (min/day) ‡	184.48 (174.40)	22.63 (76.83)	< 0.001
Computer use (min/day) ‡	90.74 (112.98)	117.35 (156.15)	0.611
TV watching (min/day) ‡	209.87 (154.22)	128.41 (141.67)	< 0.001
Total screen time (min/day) †	485,08 (268.39)	268.39 (233.18)	0.123
	**n (%)**	**n (%)**	**p-valor**
**Physical activity level** ^#^			< 0.001
Insufficiently active	187 (54.4%)	354 (65.4%)	
Active	157 (45.6%	119 (25.2%	
**Screen time** ^#^			< 0.001
<4 h	24 (7.0%)	264 (55.8%)	
>4 h	320 (93.0%)	209 (44.2%)	
**Muscle strength** ^#^			< 0.001
Adequate	331 (96.2%)	419 (88.6%)	
Inadequate	13 (3.8%)	54 (11.4%)	
**Cardiorespiratory fitnes**s^#^			< 0.001
Adequate	302 (87.8%)	314 (66.4%)	
Inadequate	42 (12.2%)	159 (33.6%)	

$\bar{x}$: mean; (dp): standard deviation; BMI: body mass index; HGS: handgrip strength; CRF: cardiorespiratory fitness; TV: television.

^†^ Independent t-test;

^‡^ Mann-Whitney U test;

^#^ Chi-Square Test

By 2017/18, the majority of girls spent less than 4 hours per day on screen-based activities (55.8%), while boys spent more than 4 hours (60.4%). Most were insufficiently active (54.3% of boys and 65.4% of girls), had adequate handgrip strength (86.7% of boys and 88.6% of girls), and demonstrated adequate cardiorespiratory fitness (both sexes at 66.4%).

**Fig 1:**
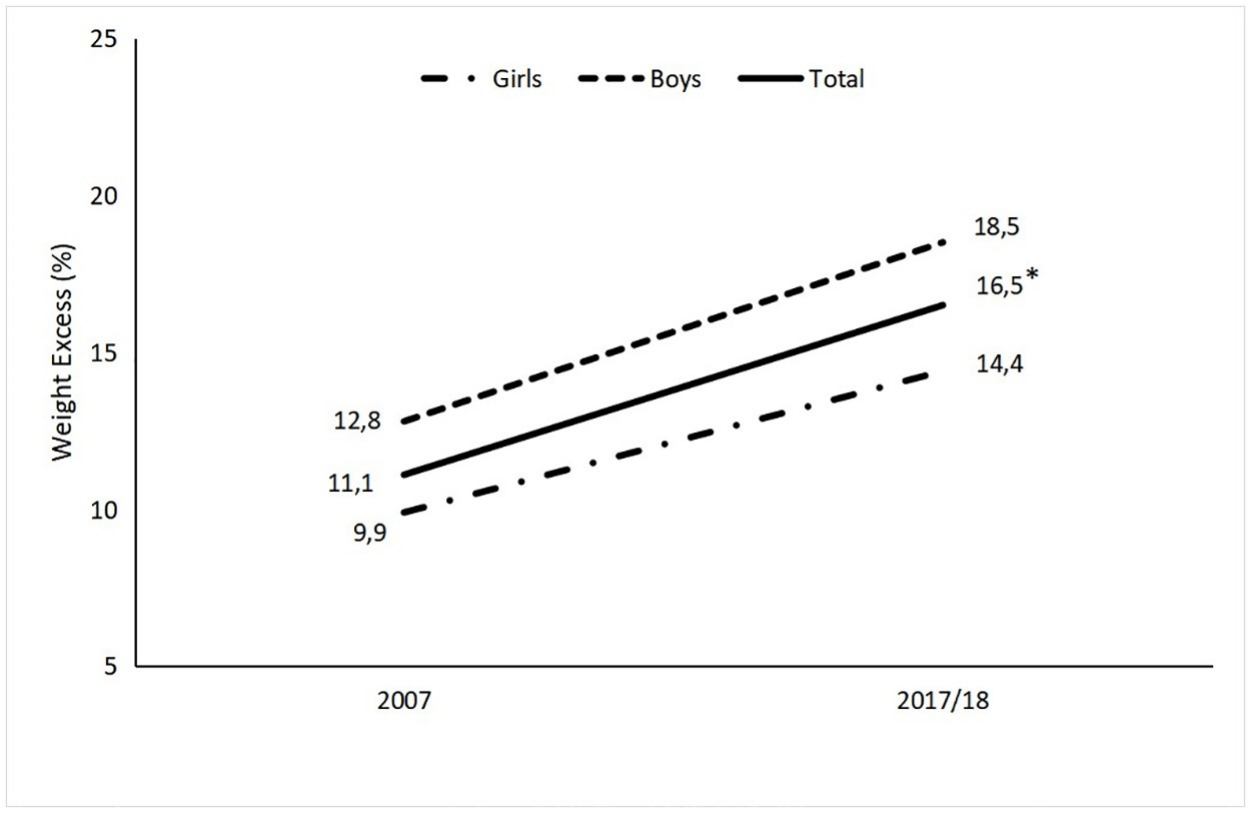
Prevalence of excess weight in adolescents in Florianópolis, Santa Catarina, Brazil, in 2007 and 2017/2018, overall and stratified by sex. * *p* < 0.05, comparison of proportions test.

Trends in excess weight overall and stratified by sex are shown in Fig 1. Comparison of 2017/2018 and 2007 data for the total sample revealed a 48.6% increase in the prevalence of excess weight (from 11.1% to 16.5%, *p* = 0.003). However, when the results were stratified by sex, there was no difference between surveys, although there was an increase of 44.5% (*p* = 0.060) in the prevalence of excess weight in boys and 45.5% (*p* = 0.055) in girls.

Table 3 shows the trends in excess weight in boys over the studied decade as a function of exposure variables. Comparative analysis of 2007 and 2017/2018 data showed that the likelihood of boys having excess weight in 2017/2018 was higher among those who spent >4 h a day on screen-based activities (OR = 1.96, 95% CI = 1.14–3.36), had inadequate muscle strength (OR = 1.77, 95% CI = 1.09–2.88), or had inadequate cardiorespiratory fitness (OR = 3.03, 95% CI = 1.25–7.38). Moreover, it was found that boys with adequate cardiorespiratory fitness were less likely to have excess weight (OR = 0.36, 95% CI = 0.17–0.77).

**Table 3:** Prevalence, temporal change (delta), and odds ratio (OD) of normal weight and excess weight according to physical activity level, screen time, muscle strength, and cardiorespiratory fitness among boys in Florianópolis, Santa Catarina, Brazil, in 2007 and 2017/2018.

Variable	% Adolescents (95% CI)		OR^†^ (95% CI)
	Normal weight	Excess weight	
*Physical activity level*			
Active			
2007	87.0 (82.7–90.4)	13.0 (8.7–16.9)	1
2017/2018	83.0 (79.7–85.7)	17.0 (13.9–19.7)	1.51 (0.79–2.89)
Delta (%)	−4.8	30.8	
Insufficiently active			
2007	87.5 (83.1–90.9)	12.5 (8.2–16.0)	1
2017/2018	80.5 (76.9–83.3)	19.5 (16.1–22.3)	1.99 (0.99–4.02)
Delta (%)	−8.0	56.0	
*Screen time*			
<4 h			
2007	78.3 (72.6–82.7)	21.7 (16.4–26.0)	1
2017/2018	82.7 (79.3–85.5)	17.3 (14.1–20.1)	0.98 (0.33–2.90)
Delta (%)	5.6	−20.3	
>4 h			
2007	88.3 (84.0–91.8)	11.7 (7.8–15.1)	1
2017/2018	80.7 (77.3–83.5)	19.3 (15.9–22.1)	1.96 (1.14–3.36)*
Delta (%)	−8.6	65.0	
*Muscle strength*			
Adequate			
2007	87.0 (84.4–89.2)	13.0 (10.5–15.2)	1
2017/2018	81.0 (77.9–83.6)	19.0 (16.0–21.6)	2.56 (0.28–23.52)*
Delta (%)	−6.9	46.2	
Inadequate			
2007	90.9 (84.4–96.1)	9.1 (3.9–13.0)	1
2017/2018	84.8 (76.6–90.9)	15.2 (7.8–22.1)	1.77 (1.09–2.88)
Delta (%)	−6.7	67.0	
Cardiorespiratory fitness			
Adequate			
2007	89.4 (84.9–92.7)	10.6 (6.9–13.7)	1
2017/2018	95.6 (93.8–97.0)	4.2 (2.6–5.6)	0.36 (0.17–0.77)*
Delta (%)	6.9	−60.4	
Inadequate			
2007	73.3 (67.1–78.1)	26.7 (21.0–31.5)	1
2017/2018	53.3 (48.9–57.0)	46.7 (42.3–50.3)	3.03 (1.25–7.38)*
Delta (%)	−27.3	74.9	

CI, confidence interval.

^**†**^Adjusted for economic level and sexual maturation stage.

*p-valor<0,05

Table 4 shows the trends in excess weight in girls over the decade as a function of exposure variables. It was observed that the chances of excess weight in girls in 2017/2018 compared with 2007 were higher among those with inadequate muscle strength (OR = 1.74, 95% CI = 1.09–2.77) and lower among those with adequate cardiorespiratory fitness (OR = 0.10, 95% CI = 0.01–0.80).

**Table 4:** Prevalence, temporal change (delta), and odds ratio (OD) of normal weight and excess weight according to physical activity level, screen time, muscle strength, and cardiorespiratory fitness among girls in Florianópolis, Santa Catarina, Brazil, in 2007 and 2017/2018.

Variable	% Adolescents (95% CI)		OR^†^ (95% CI)
	Normal weight	Excess weight	
*Physical activity level*			
Insufficiently active			
2007	88.9 (85.8–91.3)	11.1 (8.4–14.5)	1
2017/2018	85.5 (82.3–88.2)	14.5 (11.4–17.1)	1.58 (0.90–2.76)
Delta (%)	−3.8	30.6	
Active			
2007	91.7 (88.7–94.2)	8.3 (5.8–10.8)	1
2017/2018	86.1 (82.9–88.6)	13.9 (10.8–16.5)	1.60 (0.73–3.52)
Delta (%)	−6.1	67.5	
*Screen time*			
<4 h			
2007	95.8 (93.6–97.4)	4.2 (2.3–5.8)	1
2017/2018	84.5 (81.2–87.1)	15.5 (12.3–18.4)	4.24 (0.53–34.05)
Delta (%)	−11.8	269.0	
>4 h			
2007	89.7 (86.3–92.2)	10.3 (7.3–12.8)	1
2017/2018	87.1 (83.9–89.7)	12.9 (9.9–15.4)	1.44 (0.83–2.52)
Delta (%)	−2.9	25.2	
*Muscle strength*			
Adequate			
2007	90.0 (87.1–922.4)	10.0 (7.2–12.4)	1
2017/2018	85.4 (81.9–88.1)	14.6 (11.5–17.2)	1.32 (0.14–12.85)*
Delta (%)	−5.1	46.0	
Inadequate			
2007	92.3 (85.1–97.0)	7.7 (1.5–13.4)	1
2017/2018	87.0 (79.1–92.5)	13.0 (6.0–19.4)	1.74 (1.09–2.77)
Delta (%)	−5.7	68.8	
*Cardiorespiratory fitness*			
Adequate			
2007	96.7 (94.8–98.0)	3.3 (1.5–5.0)	1
2017/2018	99.7 (99.2–99.7)	0.3 (0.0–0.4)	0.10 (0.01–0.80)*
Delta (%)	3.1	−90.9	
Inadequate			
2007	42.9 (37.8–47.1)	57.1 (51.8–61.3)	1
2017/2018	57.9 (53.5–61.5)	42.1 (37.6–45.9)	0.57 (0.28–1.15)
Delta (%)	35.0	−26.3	

CI, confidence interval.

^**†**^Adjusted for economic level and sexual maturation stage.

*p-valor<0,05

## Discussion

The main findings were as follows: (1) there was a positive trend in excess weight over the 10-year study period among adolescents of both sexes, (2) boys from the 2017/2018 sample who spent more than 4 h a day on screen-based activities, and with inadequate cardiorespiratory fitness had a higher likelihood of having excess weight compared with boys from the 2007 sample, (3) adolescents of both sexes with inadequate muscle strength had greater chances of having excess weight in 2017/2018 than in 2007, and (4) adolescents with adequate cardiorespiratory fitness had lower chances of having excess weight in 2017/2018 than in 2007.

The findings are in agreement with those of international studies in the first Atlas of Childhood Obesity, published by the World Obesity Federation in 2019 [36]. The greater prevalence of obesity in boys than in girls is a common trend in most high-income and upper-middle-income countries (as is the case of Brazil). Such a trend is lower among low- and lower-middle-income countries [36, 37]. The increase in excess weight is explained by the economic (e.g., greater purchasing power), cultural, and social transitions faced by the younger population that result in a new lifestyle, inducing an increase in the consumption of ultra-processed foods, industrialized foods, soft drinks, ready-to-eat meals, and hypercaloric foods [11, 12], as well as increased sedentary behavior and low levels of physical activity [10].

Several elements can influence the development of excess weight. A plausible hypothesis for the disparity between sexes is the prolonged screen exposure. Increases in body adiposity indices coincide temporally with the increase in screen time, and less activity. Secular trend studies have shown that screen time has increased in adolescents of both sexes [17, 19]. However, prevalence rates were higher in boys when considering total screen time, whereas, in girls, prevalence rates were higher when considering television time only [17, 19]. In addition to screen time having increased among adolescents, a recent systematic review shows that 46.4% of school-aged children have screen time ≥ 2 hours per day [38].

It was found that boys who spent more than 4 h a day on screen-based activities had higher chances of having excess weight in 2017/2018 than in 2007. Excessive screen time is associated with increased overweight and obesity in adolescents [15]. The relationship between these variables can be explained by the increased food intake [39] or less healthy eating pattern during screen time (and consequently, less activity), although this was not measured in our study. Screen time greater than 4 h a day, as compared with less than 2 h a day, was associated with low vegetable consumption and high sweets consumption in girls, as well as increased soft drink consumption in adolescents of both sexes [40]. Furthermore, spending more time watching television, playing video games, and using other screen devices was positively associated with unhealthy eating patterns, including consumption of fast foods, sugary drinks, sweets, fried foods, and snacks [41], and with lower levels of physical activity [42].

Another result of the current study was that boys and girls in 2017/18 with inadequate muscle strength had a higher chance of having excess weight than those in 2007. Similar findings were reported by previous studies [43, 44]. In Brazilian adolescents, fat mass index was associated with lower handgrip strength (there was a reduction in the handgrip strength of boys and girls as the fat mass index increased), and lean body mass was associated with higher handgrip strength (there was an increase in handgrip strength as lean body mass increased) [43]. Results from a longitudinal study with Portuguese adolescents, with annual measurements for three years, showed that positive changes in handgrip strength predicted decrease in BMI values for girls and boys [44]. Even though BMI is not specific with regard to fat mass or fat-free mass [45], it is possible that the overweight adolescents in the present study have a greater amount of fat mass and a lower amount of lean body mass, which justifies the results.

Boys from 2017/2018 with inadequate cardiorespiratory fitness were more likely to have excess weight than adolescents from 2007. In agreement with these results, a previous study [4] found an inverse association between cardiorespiratory performance and excess weight (overweight and obesity) in adolescents, regardless of sex and age. It is important to highlight that both results were verified using submaximal aerobic tests, which are susceptible to errors. However, when analyzing adults with severe obesity using maximum test, it was observed that individuals with higher CRF achieved significantly greater average weight loss at 3 months and 1 year compared to those with lower CRF [46]. Furthermore, individuals who showed larger initial improvements in CRF at 3 months experienced greater weight loss at 1 year compared those with smaller changes in CRF [46]. The decline in aerobic fitness associated with an increase in overweight and obesity in adolescents was also reported in a recent systematic review [47] and in a secular trend study [48]. The extra load to be moved resulting from excess body fat may affect test performance [45], and higher BMI is related to less physical activity or increased screen time [49].

In both sex groups, adolescents in 2017/2018 with adequate cardiorespiratory fitness were less likely to have excess weight. This result can be explained by levels of physical activity, which is associated with BMI [50] and influences cardiorespiratory fitness [49]. In a study with Finnish adolescents, excess-weight individuals were less active than normal-weight individuals [50]. In Canada, cardiorespiratory fitness was better among children and adolescents who met physical activity recommendations [49]. Therefore, it is understood that adolescents with normal weight engage more in physical activity and consequently have better cardiorespiratory fitness. Another factor that may influence this result is screen time, as cardiorespiratory fitness was higher among Canadian adolescents who met screen time recommendations, and screen times greater than 4 h a day were associated with excess weight [49].

This study has some limitations: i) Questionnaires on economic status, physical activity and screen time were self-report, which may lead to errors associated with filling and interpretating the questionnaire. Furthermore, the possibility that adolescents provided socially desirable answers instead of real answers cannot be ruled out. However, given the ease and low cost of administration, these instruments are widely used in epidemiological studies; ii) CRF was measured using a submaximal test; iii) Although muscle strength, as measured by the handgrip strength test, is an indicator of overall health, the results vary depending on the population studied. Individuals with greater height and body mass tend to achieve higher results. Additionally, since it involves gripping strength, the results can be influenced by the activities performed by the individual being assessed. Individuals engaged in manual tasks that require greater upper limb resistance will likely achieve higher results compared to those who do not engage in such activities. Moreover, handgrip strength may not necessary reflect overall strength, such as strength in the lower limbs or trunk; iv) The dietary intake of adolescents was not assessed; v) The sample was composed exclusively of adolescents from the public school system, a factor that limits inferences about adolescents from private schools. Therefore, the findings of the present study cannot be extrapolated to a large population, to individuals who attend private schools, or to those who are not enrolled in the school system. Some of the strengths of this study include the use of repeated measures and the possibility of comparison, as the same protocol was used in both surveys on excess weight for screen time, muscle strength, and cardiorespiratory fitness assessment.

## Conclusions

A positive trend in the prevalence of excess weight was found among adolescents between 2007 and 2017/2018. Boys who spent more than 4 h a day on screen-based activities, those with adequate muscle strength, and those with inadequate cardiorespiratory fitness were more likely to have excess weight in 2017/2018, whereas adolescents with adequate cardiorespiratory fitness had lower chances of having excess weight. Girls in 2017/2018 with adequate handgrip strength had higher chances of having excess weight and girls with adequate cardiorespiratory fitness had lower chances of having excess weight.

These results can contribute to guiding actions and public policies aimed at mitigating obesity in adolescence. They may also promote the integration of different sectors and professionals with the aim of developing strategies for the promotion of a healthy lifestyle and disease prevention.
